# Optimal entrainment of heterogeneous noisy neurons

**DOI:** 10.3389/fnins.2015.00192

**Published:** 2015-05-29

**Authors:** Dan Wilson, Abbey B. Holt, Theoden I. Netoff, Jeff Moehlis

**Affiliations:** ^1^Department of Mechanical Engineering, University of California, Santa BarbaraSanta Barbara, CA, USA; ^2^Graduate Program in Neuroscience, University of MinnesotaMinneapolis, MN, USA; ^3^Department of Biomedical Engineering, University of MinnesotaMinneapolis, MN, USA

**Keywords:** entrainment, noisy neurons, noisy oscillators, uncertainty, optimal control theory

## Abstract

We develop a methodology to design a stimulus optimized to entrain nonlinear, noisy limit cycle oscillators with uncertain properties. Conditions are derived which guarantee that the stimulus will entrain the oscillators despite these uncertainties. Using these conditions, we develop an energy optimal control strategy to design an efficient entraining stimulus and apply it to numerical models of noisy phase oscillators and to *in vitro* hippocampal neurons. In both instances, the optimal stimuli outperform other similar but suboptimal entraining stimuli. Because this control strategy explicitly accounts for both noise and inherent uncertainty of model parameters, it could have experimental relevance to neural circuits where robust spike timing plays an important role.

## 1. Introduction

Precise timing of synchronized oscillators is an important aspect of many biological functions. For example, entrainment of circadian oscillators to a 24-h light-dark cycle is necessary in nearly all organisms for the maintenance of rhythmic physiological function (Winfree, 2001; Golombek and Rosenstein, 2010); irregularities in circadian regulation can contribute to a wide variety of diseases (Canaple et al., 2003; Klerman, 2005; Takeda and Maemura, 2011). Furthermore, pancreatic cells can be entrained to periodic variations in blood glucose levels, synchronizing the activity of the insulin secreting cells (Bertram et al., 2007; Pedersen et al., 2013). Also, synchronized patterns of firing neurons give rise to macro scale brain rhythms which are thought to be relevant to cognition and perception (Buzsáki and Draguhn, 2004; Jacobs et al., 2007; Lakatos et al., 2008; Ainsworth et al., 2012), and in specific examples lack of synchrony can contribute to hearing loss in animals (Wang and Manis, 2006; Henry and Heinz, 2012).

Promoting synchrony by means of entrainment to an external stimulus could facilitate physiological processes where synchronization is important. Optimal control frameworks can be used to achieve specific control objectives where timing is the control variable and entrainment is the goal (Kiss et al., 2007; Harada et al., 2010; Zlotnik et al., 2013). However, these approaches fall short when heterogeneity in the oscillator properties is large with respect to intensity of the entraining stimulus. Furthermore, most optimal control techniques cannot explicitly account for strong noise in the system, which is often inherent in biological systems (especially in neurons Tuckwell, 2005; Ermentrout and Terman, 2010) degrading the efficacy of an optimal stimulus.

In this work, we use standard phase reduction techniques to model the response of an oscillator to external perturbations. A phase response curve (PRC) is fit to the phase advance as a function of the phase at which the stimulus is applied (Kuramoto, 1984; Winfree, 2001; Izhikevich, 2007). Phase reduction techniques are advantageous because they characterize much of the system's input-output function without the full nonlinear dynamical equations. Using the PRC, we are able to derive sufficient conditions for a stimulus to entrain a noisy, heterogeneous ensemble of phase oscillators. Furthermore, once the sufficient conditions are identified, we can then design efficient external stimuli for entrainment. This strategy does not require the explicit properties of any single oscillator, but only requires the bounds within which all the oscillator's PRCs and natural frequencies must be contained. Using stochastic averaging techniques (Freidlin and Wentzell, 2012), we can design a stimulus which creates a potential well of minimum depth near the in-phase solution between a nominal oscillator and any other oscillator within the heterogeneous ensemble. Deeper potential wells will be harder to escape from when the noise is present in the system, ensuring entrainment. Unlike other approaches we have used (Wilson and Moehlis, 2014a), the innovation proposed in this strategy is that the control design explicitly accounts for noise and heterogeneity present in the biological system.

We test the efficacy of the optimal synchronizing stimulus on coupled phase oscillator models compared to other entraining stimuli. We then design optimal stimulus waveforms from previously collected PRCs and test the resulting optimized stimulus *in vitro* on pyramidal neurons from the CA1 region of the hippocampus. The organization of this paper is as follows. In Section 2 we derive the necessary framework for designing stimuli to entrain a heterogeneous population of oscillators. In Section 3, we apply this control strategy to a numerical model of a population of heterogeneous phase oscillators. In Sections 4 and 5 we provide experimental methods and results, respectively, for entrainment of *in vitro* neurons, and finally in Section 6 we discuss our findings and make concluding remarks.

## 2. Efficiently maximizing the depth of the potential well

Consider the following deterministic phase oscillator

(1) $${\dot{\theta }}_{1}=\omega_{0}+Z(\theta_{1})\epsilon u(t).$$

Here, θ_1_ ∈ [0, 2π) is the phase of a nominal reference oscillator with natural frequency ω_0_ and period *T* = 2π/ω_0_, *Z*(θ) is the phase response curve, and *u*(*t*) is an external input, and 0 < ϵ ≪ 1. Note that we assume that ϵ is small enough so that higher order noise terms are negligible (c.f. Ly and Ermentrout, 2009; Ermentrout et al., 2011). A second noisy oscillator, θ_2_, whose parameters are not fully known, can be represented as follows:

(2) $$\begin{array}{l} {\dot{\theta }}_{2}=\omega_{0}+\Delta \omega +[Z(\theta_{2})+\Delta Z(\theta_{2})]\epsilon u(t)+[Z(\theta_{2})\\ \;+\;\Delta Z(\theta_{2})]\epsilon \eta (t). \end{array}$$

**Figure 1:**
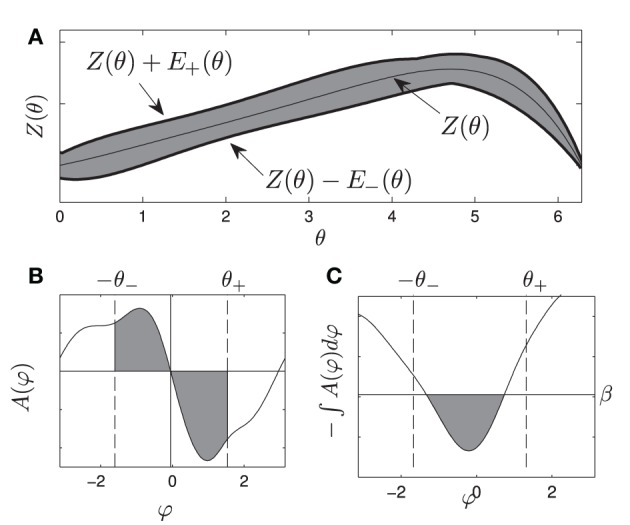
**(A)** gives a visual representation of the uncertainty allowed in the phase response curve from Equation (1), i.e., any PRC that can be drawn inside the shaded region is allowable. **(B)** shows an example of *A*(ψ). gives a visual representation of the requirements Equations (10) and (11), for any oscillator with any allowable PRC, the area of each shaded region in **(B)** must be at least β. If this is the case, the resulting potential well in **(C)** will be at least β high by the time ψ is smaller than −θ_−_ or larger than θ_+_.

This function is illustrated in Figure 1A. The variable Δω ∈ [−ϵ ω_−_, ϵ ω_+_] represents some uncertainty in the natural frequency, Δ*Z* represents uncertainty in the phase response curve, and η(*t*) = 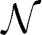
(0, 1) is i.i.d zero mean white noise with variance 1. However, for the population, we can determine bounds for the range of the PRCs −*E*_−_(θ) ≤ Δ*Z*(θ) ≤ E_+_(θ) with strictly nonnegative functions *E*_−_(θ) and *E*_+_(θ). Intuitively, θ_1_ in Equation (1) represents the nominal parameters of an oscillator while θ_2_ in Equation (2) accounts for the uncertain terms, which may not be fully known, as well as noise that might be present in the system. Alternatively, θ_2_ can represent a range of properties for a heterogeneous population of oscillators.

We assume that the reference oscillator is entrained to the external stimulus so that

(3) $$\int_{0}^{T}\left[\omega_{0}+Z(\theta_{1})\epsilon u(t)\right]dt=2\pi .$$

Therefore, to guarantee that the noisy, uncertain oscillator is also entrained, our goal is to design *u*(*t*) such that the phase difference between the two oscillators is small. Defining ϕ = θ_2_ − θ_1_, we may write

(4) $$\begin{array}{l} \dot{\phi }=\Delta \omega \;+\;\left[Z(\theta_{1}+\phi )-Z(\theta_{1})+\Delta Z(\theta_{1}+\phi )\right]\epsilon u(t)\\ \;+\;\left[Z(\theta_{1}+\phi )+\Delta Z(\theta_{1}+\phi )\right]\epsilon \eta (t). \end{array}$$

Here, ϕ gives the phase difference between the nominal oscillator and the noisy, unknown oscillator so that when ϕ = 0, both oscillators are in phase. Asymptotically expanding θ_1_ in powers of ϵ yields

(5) $$\theta_{1}=\theta_{1}^{\left(0\right)}(t)+\epsilon \theta_{1}^{\left(1\right)}(t)+\epsilon^{2}\theta_{1}^{\left(2\right)}(t)+\ldots$$

Note that all terms of Equation (4) are 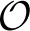
(ϵ), which implies that θ^(0)^_1_(*t*) = θ_1_(0) + ω_0_*t* so that θ_1_(*t*) = θ_1_(0) + ω_0_*t* + 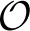
(ϵ). For convenience, we take θ_1_(0) = 0, but note that the analysis to follow could still be performed for θ_1_(0) ≠ 0. Substituting Equation (5) into Equation (4) and Taylor expanding terms of the form *Z*(·) in powers of ϵ yields

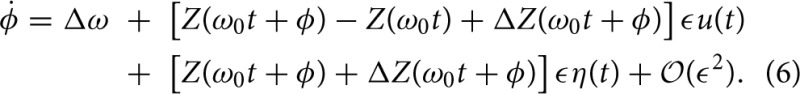



Through stochastic averaging (Zhu, 1988; Freidlin and Wentzell, 2012) in the limit of small ϵ, we can approximate ϕ in Equation (4) by φ where

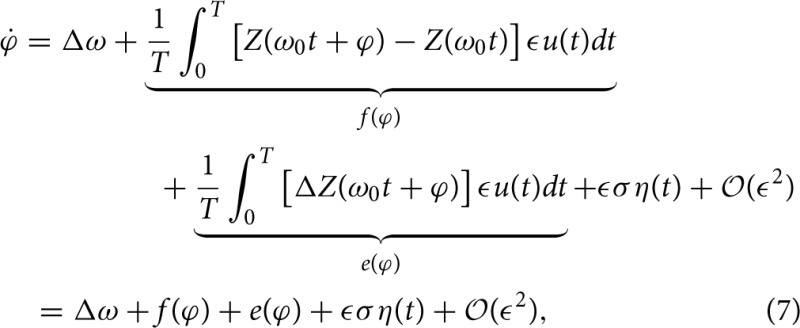



where *f*(ψ) represents the known part of Equation (4), *e*(ψ) represents the uncertain part of Equation (4), and

(8) $$\begin{array}{l} \sigma^{2}=\frac{1}{T}\int_{0}^{T}{\left[Z(\omega_{0}t+\varphi )+\Delta Z(\omega_{0}t+\varphi )\right]}^{2}dt\\ \;=\frac{1}{T}\int_{0}^{T}{\left[Z(\omega_{0}t)+\Delta Z(\omega_{0}t)\right]}^{2}dt \end{array}$$

determines the strength of the noise. Note that equivalence in Equation (8) comes from periodicity in the PRC. The equation for the probability distribution function associated with Equation (7) is given by the Fokker-Planck equation (Gardiner, 2004)

(9) $$\frac{\partial \rho }{\partial t}=-\frac{\partial }{\partial \varphi }\left[A(\varphi )\rho (t,\varphi )\right]+\frac{1}{2}\frac{\partial^{2}}{\partial \varphi^{2}}\left[B\rho (t,\varphi )\right],$$

where ρ(*t*, φ) is a probability density function, *A*(φ) = Δω + *f*(φ) + *e*(φ) and *B* = ϵ^2^ σ^2^. From this perspective, it is not possible to maintain indefinite entrainment of the noisy neuron, because there is always a chance that noise in the system could push the neuron arbitrarily far from φ ≈ 0. However, to reduce the likelihood of this event, the problem of entraining a noisy neuron to a periodic stimulus can be viewed as maximizing the average escape time from φ = 0 over a potential barrier. From this perspective, for an oscillator whose probability density obeys Equation (9), ∫ − *A*(φ) *d*φ can be viewed as a potential function. Therefore, our goal is to design a stimulus so that there is a potential barrier with a minimum near φ ≈ 0. The escape time from this barrier can be expected to be proportional to the exponential of the height of the potential barrier (Gardiner, 2004).

To maximize the escape time we want to design a stimulus *u*(*t*) such that the change in a potential trough near φ = 0 and a potential peak located at either φ = θ_+_ > 0 or φ = − θ_−_ < 0 is greater than or equal to β. This requirement can be stated as:

(10) $$\int_{0}^{\theta_{+}}-A(\varphi )d\varphi \geq \beta ,$$

(11) $$\int_{0}^{-\theta_{-}}-A(\varphi )d\varphi \geq \beta .$$

Here β can be thought of as the minimum height required for the potential well near φ = 0. Alternatively, β in Figure 1B represents the minimum area in each shaded region required to produce a potential well with a size of at least β between −θ_−_ and θ_+_, as in Figure 1C.

Recall that to leading order ϵ, *A*(φ) = Δω + *f*(φ) + *e*(φ), substituting these terms from Equation (7) into Equation (10) gives

(12) $$\begin{array}{l} \int_{0}^{\theta_{+}}[\Delta \omega +\frac{1}{T}\int_{0}^{T}\left[Z(\omega_{0}t+\varphi )-Z(\omega_{0}t)\right]\epsilon u(t)dt\\ \;+\frac{1}{T}\int_{0}^{T}\left[\Delta Z(\omega_{0}t+\varphi )\right]\epsilon u(t)dt]d\varphi \leq -\beta ,\\ \frac{1}{T}\int_{0}^{T}[\Delta \omega \theta_{+}+\left(\int_{0}^{\theta_{+}}\left[Z(\omega_{0}t+\varphi )-Z(\omega_{0}t)\right]d\varphi \right)\epsilon u(t)\\ \;+\left(\int_{0}^{\theta_{+}}\left[\Delta Z(\omega_{0}t+\varphi )\right]d\varphi \right)\epsilon u(t)]dt\leq -\beta . \end{array}$$

By noting that

(13) $$\begin{array}{l} \int_{0}^{\theta_{+}}-E_{-}(\omega_{0}t+\varphi )d\varphi \leq \int_{0}^{\theta_{+}}\Delta Z(\omega_{0}t+\varphi )d\varphi \leq \\ \;\int_{0}^{\theta_{+}}E_{+}(\omega_{0}t+\varphi )d\varphi , \end{array}$$

we can use this inequality in Equation (12) to conclude that if

(14) $$\frac{1}{T}\int_{0}^{T}\left[\epsilon \omega_{+}\theta_{+}+\left[g_{+}(t)+E_{p}(t,u)\right]\epsilon u(t)\right]dt\leq -\beta ,$$

where

(15) $$E_{p}(t,u)=\{\begin{array}{ll} \int_{0}^{\theta_{+}}E_{+}(\omega_{0}t+\varphi )d\varphi & \text{if }u\geq 0,\\ \int_{0}^{\theta_{+}}-E_{-}(\omega_{0}t+\varphi )d\varphi & \text{if }u<0, \end{array}$$

(16) $$g_{+}(t)=\int_{0}^{\theta_{+}}\left[Z(\omega_{0}t+\varphi )-Z(\omega_{0}t)\right]d\varphi ,$$

then Equation (12) and hence Equation (10) must also hold. Using similar logic, (i.e., manipulating Equation 11 so it is in the same form as Equation 12, then using the inequality Equation 13) we can conclude that

(17) $$\frac{1}{T}\int_{0}^{T}\left[-\epsilon \omega_{-}\theta_{-}+[g_{-}(t)+E_{m}(t,u)]\epsilon u(t)\right]\geq \beta ,$$

where

(18) $$E_{m}(t,u)=\{\begin{array}{ll} \int_{-\theta_{-}}^{0}-E_{-}(\omega_{0}t+\varphi )d\varphi & \text{if }u\geq 0,\\ \int_{-\theta_{-}}^{0}E_{+}(\omega_{0}t+\varphi )d\varphi & \text{if }u<0, \end{array}$$

(19) $$g_{-}(t)=\int_{-\theta_{-}}^{0}\left[Z(\omega_{0}t+\varphi )-Z(\omega_{0}t)\right]d\varphi ,$$

is a sufficient condition for Equation (11) to be true.

Thus, the control objective of creating a potential well that is at least β deep can be accomplished by designing a stimulus such that Equations (14) and (17) are satisfied. We can solve for an energy-optimal stimulus which accomplishes this goal with a Hamilton-Jacobi-Bellman (HJB) approach (Kirk, 1998) by defining the auxiliary state vector *z* such that

(20) $$\dot{z}=\left[\begin{array}{c} \dot{a}\\ \dot{b}\\ \dot{\theta } \end{array}\right]=\left[\begin{array}{c} \frac{1}{T}(\epsilon \omega_{+}\theta_{+}+\left[g_{+}(t)+E_{p}(t,u)\right]\epsilon u(t))\\ \frac{1}{T}(-\epsilon \omega_{-}\theta_{-}+[g_{-}(t)+E_{m}(t,u)]\epsilon u(t))\\ \omega_{0}+Z(\theta )\epsilon u(t) \end{array}\right],$$

where *a* and *b* are auxiliary variables which come from the constraints Equations (14) and (17), respectively. The variable θ is included so that we can specify the end point conditions

(21) θ(0)=0  and  θ(T)=2π,

requiring the noiseless oscillator with nominal properties, θ_1_, to be perfectly entrained to the external stimulus. Hence, the true noisy uncertain oscillator θ_2_ will also be entrained to the external stimulus when it is inside the potential well at φ ≈ 0.

For the initial state *z* = [0, 0, 0]^*T*^, the energy optimal stimulus will minimize

(22) $$J(z,u(t))=\int_{0}^{T}u^{2}dt+q(z(T)),$$

where ∫*^T^*_0_ *u*^2^*dt* represents the energy consumed by the stimulus, and *q*(*z*(*T*)) is an end-point cost function where *q*(*z*([*a*(*T*), *b*(*T*), θ(*T*)]^*T*^)) is small for final states states where *a*(*T*) ≤ −β, *b*(*T*) ≥ β and θ(*T*) = 2π, and large otherwise. This endpoint cost is chosen to give a prohibitive penalty if the stimulus *u*(*t*) does not satisfy the required constraints Equations (14), (17), and (21). The energy optimal stimulus, u^*^(*t*), can be found with standard HJB techniques, and a related example is given in greater detail in Wilson and Moehlis (2014a).

As a final note, we examine how the optimal stimulus changes when the natural frequency ω_0_ changes and both θ_−_ and θ_+_ = 0. To this end, suppose that we have already solved Eqaution (22) for the optimal stimulus u^*^(*t*) when the natural frequency is ω_0_ = 2π/*T_o_*. Suppose that u^*^(*t*) = ϵ_1_*u_o_*(*t*), to leading order, the requirement Equation (3) is

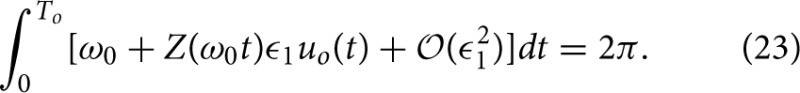



Now consider a different natural frequency ω_1_ = 2π/*T*_1_. The requirement Equation (3) is

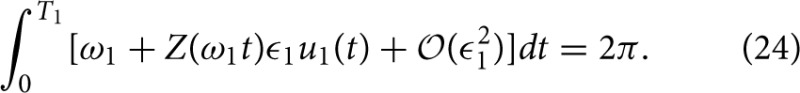



Changing variables so that $\tau =\frac{T_{o}}{T_{1}}t$ we can rewrite Equation (24) as

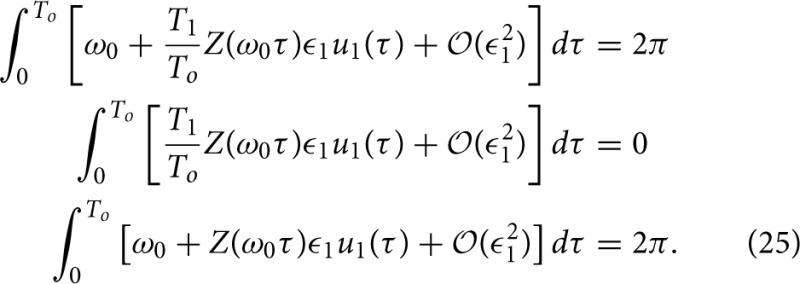



Notice that Equation (25) is equivalent to Equation (23). One can verify that using the same change of variables, the constraints Equations (14) and (17) can be made identical for the two natural frequencies. Thus, the auxiliary state dynamics $\dot{z}={\left[\dot{a},\dot{b},\dot{\theta }\right]}^{T}$ will be the same, which implies $u_{0}(t)=u_{1}(\tau )=u_{1}\left(\frac{T_{o}}{T_{1}}t\right)$, which is a useful property from an experimental perspective.

## 3. Numerical results

For numerical validation of the theory, we apply the methods from Section 2 to a large population of *N* = 1000 noisy phase oscillators,

(26) $$\theta_{j}=\omega_{j}+Z_{j}(\theta_{j})u(t)+\eta_{j}(t),\;j=1,\ldots ,N.$$

**Figure 2:**
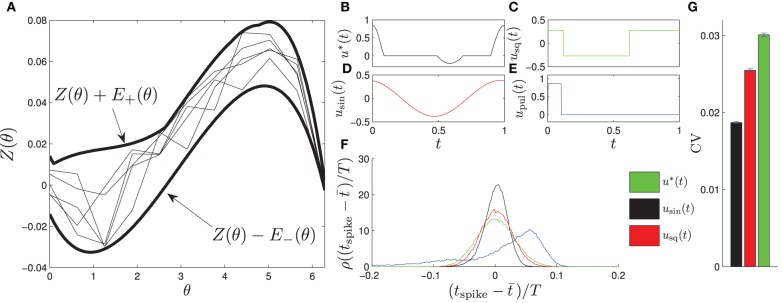
**(A)** shows the envelope in which the PRCs of each of the phase oscillators fit, shown as thick lines. Examples of individual PRCs are shown as thin lines. The optimal entraining stimulus u^*^ and three other stimuli *u*_sin_, *u*_sq_, and *u*_pul_ (shown in **B–E**) are applied periodically to test their entrainment of the noisy ensemble (Equation 26). **(F)** shows a probability density of spike times relative to the phase of the entraining stimulus, ρ(*t*_spike_ − *t*), where *t* is the average spike time. We find that the optimal stimulus yields a significantly tighter distribution of spike times, as reflected in the coefficient of variation shown in **(F)** calculated from their distribution of spike times. We note that the coefficient of variation for the pulsed stimulus is 0.091, and do not show it on the graph because it is much larger than the other values.

Here, *Z_j_*(θ) is constrained to be within the envelope from the left panel of Figure 2, which is determined from experimental calculations of pyramidal neurons from the CA1 region of the hippocampus. We choose the envelope in this way to mimic the *in vitro* experiments performed in the sections to follow. To determine the PRC for each oscillator, 11 control points are randomly chosen at equally spaced intervals within the envelope, and *Z_j_*(θ) is linearly interpolated between the control points. Example PRCs are shown in Figure 2. We note that this envelope is relatively large, so the variance in ω_*j*_ is taken to be relatively small with ω*_j_* = ω_0_ + Δω, with Δω ∈ [−0.004 ω_0_, 0.004 ω_0_] chosen from a uniform distribution. A larger variance in the natural frequencies could be chosen if the envelope of possible PRCs is smaller. We also take $n_{j}(t)=\sqrt{0.05\nu_{j}}$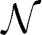 (0, 1) to be i.i.d. zero mean white noise with variance 0.05 ν_*j*_, where $\nu_{j}=\frac{1}{2\pi }\int_{0}^{2\pi }Z_{j}^{2}(\theta )d\theta$.

For calculation of the optimal stimulus, we take the nominal PRC to be the average of the PRCs taken from multiple CA1 pyramidal cells, which is close to the average between the top and bottom curves in Figure 2A. We take θ_+_ = θ_−_ = 0.94, β = 10^−4^, and *T* = 24 ms. The optimal control is shown as u^*^(*t*) in Figure 2B with the other applied stimuli shown in Figures 2C–E. Generally, the optimal control seeks to apply a positive (resp. negative) stimulus when the slope of the PRC is negative (resp. positive) and when the derivative is large in magnitude relative to the size of the envelope. For example, a large positive stimulus is given near the end of the cycle when the derivative is very negative and the uncertainty is relatively small; conversely, no stimulus is given near the beginning of the cycle when the slope is small in magnitude and the uncertainty is high. We also compare the resulting entrainment when using the optimal stimulus to the entrainment using a sine wave, square wave, and square pulse, *u*_sin_, *u*_sq_, and *u*_pul_, respectively, each using an equivalent amount of power. We simulate the system (Equation 26) for 60*T* with *u*(*t*) taken to be one of these four stimuli applied periodically and report the time at which the cells spike (i.e., cross θ = 2π) as a probability distribution ρ relative to the phase of the periodic stimulus. Results are shown in Figure 2F. We find that the optimal stimulus gives the sharpest distribution of spike times. The coefficient of variation (CV) from a sample of 60,000 spike times are reported in Figure 2G with error bars representing a 95 percent confidence interval assuming that the spike time distribution is well approximated by a normal distribution.

## 4. Experimental methods

To test the efficacy of the optimized stimulus waveform in a biological system, we designed stimulus waveforms to entrain hippocampal CA1 pyramidal neurons in a brain slice preparation. PRCs were first measured from several pyramidal neurons to estimate the variability in the PRC waveform. Then, optimized stimulus waveforms were designed and applied to neurons using patch clamp recording techniques. All experimental procedures were performed following guidelines from Research Animal Resources of the University of Minnesota and approved by the Institutional Animal Care and Use Committee.

### 4.1. Electrophysiology recordings

Hippocampal brain slices were prepared from Sprague Dawley rats aged 14–21 days old. Rats were deeply anesthetized using isoflurane before decapitation and extraction of the brain. Following extraction, the brain was chilled in artificial cerebral spinal fluid (aCSF) composed of (in mM): 125 NaCl, 25 NaHCO_3_, 11 D-glucose, 3 KCl, 1.25 NaH_2_PO_4_, 2 CaCl_2_, and 1 MgCl_2_. Transverse slices of the hippocampus were sectioned 400 μm thick using a Vibratome. Slices were oxygenated with 95% O_2_ and 5% CO_2_ and incubated at 37 °C for at least 1 h. Slices were visualized using differential interference contrast optics (Olympus, Center Valley, PA) while in a chamber with circulating aCSF. Patch-clamp electrodes (3–6 MΩ) were pulled from borosilicate glass (P-97 micropipette puller; Sutter Instrument) and filled with intracellular recording fluid composed of (in mM): 120 K-gluconate, 10 HEPES, 1 EGTA, 20 KCl, 2 MgCl_2_, 2 Na_2_ATP, and 0.25 Na_3_GTP. Recordings from whole-cell patch clamped CA1 pyramidal neurons in the hippocampus were made using a current-clamp amplifier (MultiClamp 700B; Axon Instruments, Molecular Devices, Sunnyvale, CA). Data were collected using the Real-Time eXperimental Interface (RTXI) software publicly available (www.rtxi.org) and sampled at 5 kHz.

### 4.2. Estimating PRCs from neurons

**Figure 3:**
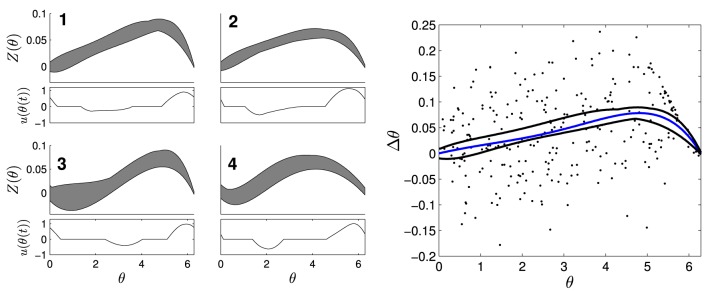
**Four envelopes with separate optimal waveforms were used to determine which stimuli to use on a given cell**. The left panels show envelopes in gray with corresponding optimal stimulus waveforms directly below. On the right panel, a PRC (blue) calculated from individual measurements of Δθ/*Q* (dots) from a CA1 pyramidal neuron fits within the black curves of envelope 1.

To estimate PRCs from CA1 pyramidal neuron neurons, stimuli were applied at different phases of the neuron's interspike interval and deviations from the unperturbed period was measured. PRCs were estimated as previously described in Nabi et al. (2013), c.f. Netoff et al. (2012). Briefly, short-duration (0.02–0.4 ms) current pulses (300–400 pA) were injected into the periodically firing neuron through the patch clamp electrode to elicit a significant phase change without inducing an action potential. Each data point was obtained by stimulating at a random phase θ, and measuring the change in spike time with the resulting value *Z*(θ) equal to Δθ/*Q*, where Δθ is the change in phase and *Q* is the charge injected by the pulse. Constant drive or an oscillatory input to these neurons causes them to fire periodically. To compensate for drift in the neuron's natural firing rate over the experiment, a proportional-integral (PI) controller was used to adjust the current applied to the neuron slowly to maintain the neuron at a target firing rate (Miranda-Dominguez et al., 2010). Spike advance as a function of the stimulus phase was fit with a low order polynomial constrained to zero at the beginning and end of the phase by minimizing least squares error (Matlab's fminsearch). Examples of PRCs are shown in Figure 3. We note that the waveform optimization presented in Section 2 requires the mean phase advance estimated by the PRC to be within the envelope, but the phase advance on any particular cycle can be outside the envelope, due to noise.

### 4.3. Stimulus waveform

PRCs from ten CA1 pyramidal cells, collected for previous experiments under similar conditions described here (Miranda-Dominguez and Netoff, 2013; Nabi et al., 2013), were used to design optimal stimulus waveforms. The PRCs used for optimization had slightly different shapes, so four envelopes and their corresponding stimulus waveforms were calculated; shown in Figure 3. The parameters used in this optimization were θ_+_ = θ_−_ = 1.89, and β = 0.0024. We assume that we have direct control over the natural frequency of the neuron with the PI controller and set Δω = 0. Each optimal waveform was defined by an envelope containing all PRCs within that group of cells. For each envelope, one optimal and two suboptimal stimulus waveforms with equal energy were generated. The suboptimal waveforms were created by either inverting and time-shifting the optimal waveform, or by stretching out the positive portion of the optimal waveform and renormalizing to preserve the total energy. For the *in vitro* experiments, whole-cell patch clamp recordings were made from CA1 pyramidal neurons. For each cell, Matlab was used to determine which envelopes the measured PRC fit within. The stimuli for the envelope with the best margins for each cell were applied as current through the patch clamp electrode. For some cells, PRCs fit within multiple envelopes, and all were tested if possible. Each of the three waveforms were applied continuously for at least 30 s to a few minutes. The stimulus waveform was applied at the target frequency of the neuron, set at 10 Hz using the PI controller, for the duration of the experiment. The peak-to-peak amplitude of the waveform was less than 1 nA. The sequence in which the waveforms were applied was selected at random. In most cases the PI controller to hold the neuron at the target firing rate was on while the stimuli were being applied, however in a few cases the PI controller was turned off to ensure it was not affecting the synchrony. The amplitude of stimulation was chosen so that the stimulus waveform could be seen in the baseline membrane potential without eliciting a spike. The experimenter was blinded to which stimulus was optimal until after completion and analysis of all experiments.

### 4.4. Entropy estimation

Entropy values calculated from spike density histograms (**Figure 5**) were used to compare how well a stimulus entrained the neuron. Data were analyzed using Matlab. For entropy calculations, we subdivide phases into *B* equally spaced bins and denote *P*(i) as the probability that a spike occurs in bin *i*. An entropy bias term was used to correct for the different number of spikes in each trial (Roulston, 1999):

(27) $${\text{Entropy}}_{\text{bias}}=\frac{B-1}{2N},$$

where *N* is the total number of spikes. To calculate the unbiased normalized entropy measure from each spike density histogram, the entropy, accounting for the bias, was normalized by the maximum possible entropy:

(28) $$\text{Entropy}=\frac{\sum_{i=1}^{B}P(i)\ln P(i)-{\text{Entropy}}_{\text{bias}}}{B\ln\frac{1}{B}}.$$

The standard error of the entropy was estimated as follows (Roulston, 1999):

(29) $$\text{SEM}=\sqrt{\frac{1}{N}\sum_{i=1}^{B}(\ln(1-P(i))+\text{Entropy}{)}^{2}P(i)(1-P(i))}.$$

Statistical comparisons between entropy values were made using the Student's *t*-test, and *p* < 0.05 were considered significant.

## 5. Experimental results

**Figure 4:**
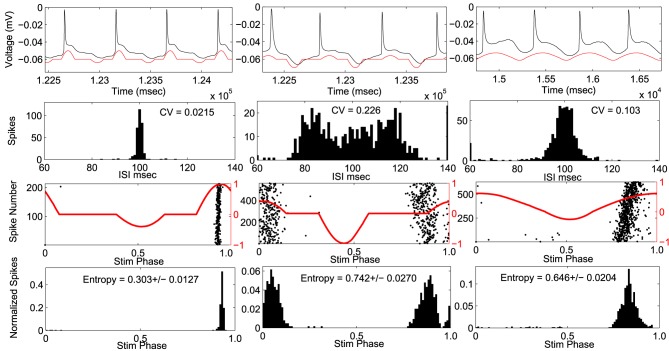
**Example cell using envelope 3**. Response to optimal stimulus is plotted in left column and two suboptimal stimuli applied in right columns. Top Row: voltage trace (black) and applied stimulus waveform (red). Second row: Histograms of inter-spike-intervals. Coefficient of variation (CV) values are indicated. Third row: phase of the stimulus at each action potential (black dots) with stimulus waveform (red). Bottom row: spike density histogram with respect to stimulus phase. Entropy values ± SEM are indicated.

Stimulus waveforms were applied to ten CA1 pyramidal neurons. An example cell can be seen in Figure 4. The PRC from this example neuron fit within envelope 3 best. For each stimulus the coefficient of variation of the interspike intervals, and the entropy of the spike times with respect to the phase of the stimulus waveform was measured. In this cell, the optimal stimulus waveform resulted in the lowest coefficient of variation in the interspike intervals, indicating that the cell fires more periodically than with the suboptimal waveforms. Furthermore, the optimal stimulus waveform had the lowest entropy of spike times with respect to the stimulus phase, indicating that the neurons phase locked to the optimal stimulus better than the suboptimal stimulus waveforms.

**Figure 5:**
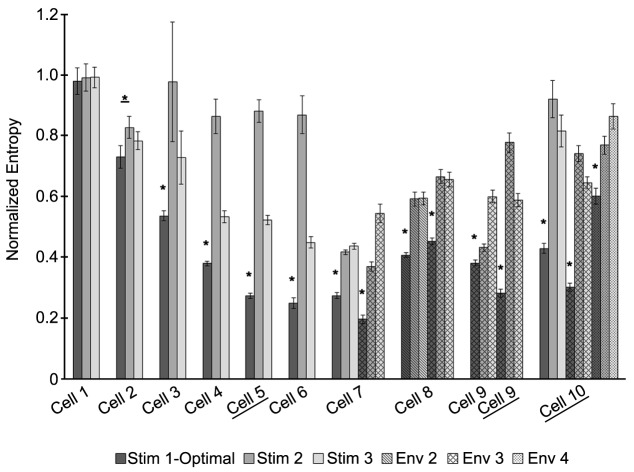
**The optimal stimulus waveform is significantly better at entraining neurons to the stimulus across cells**. Entropy values for each stimulus applied are shown for 10 cells. Some cells had stimuli from more than one envelope applied. Envelopes are indicated by different patterns, with envelope 1 being the solid fill. For each envelope, three stimuli were applied: the waveform optimized for entraining the neuron (dark gray) and two sub-optimal waveforms (gray and white). Certain cells did not have the PI controller on to control the firing rate of the neuron (underlined). Significant differences between the optimal stimulus waveform and the other waveforms at *p* < 0.05 are indicated by ^*^.

The optimal stimulus was the best, compared to the suboptimal waveforms with the same amount of power, at entraining across all recorded neurons where the stimulus had a significant effect on the entropy (Figure 5). Figure 5 shows the entropy values for stimuli across all cells. Stimuli from envelope 1 were applied to seven cells. For six out of the seven cells, the entropy values for the optimal waveform were significantly lower (*p* < 0.05) than the non-optimal waveforms, as tested with a Student's *T*-test. For cell number 1, the entropy remained high across all stimuli without any noticeable effect from any of the waveforms, perhaps because the stimulus amplitude was too low. Stimuli from envelope 2 were applied to one cell, from envelope 3 were applied to four cells, and from envelope 4 were applied to one cell. For each of these cells, the entropy values were significantly lower for the optimal waveform than the suboptimal waveforms. We conclude that the optimal stimulus waveform was the most effective at entraining the neurons to the stimulus. In three cells experiments were done without the PI controller to control the firing rate to confirm that the PI controller was not affecting the findings; the results in these cells were consistent with the experiments done with the PI controller.

## 6. Discussion

In this paper we have developed an approach to generate an optimal stimulus waveform to achieve entrainment of a noisy, heterogeneous group of phase oscillators. The waveforms were tested in computational *in silico* models and *in vitro* neurons. The optimal waveform for entrainment was determined by maximizing the average escape time from a potential well near the entrained solution for any oscillator within the ensemble. We note that this optimal control methodology only requires bounds, Equations (14) and (17), in which all oscillators in the population must be contained. While a stimulus waveform may be designed to entrain a particular oscillator in the population, the resulting stimulus may not entrain another oscillator within the population resulting in poor entrainment overall. Our method uses a worst case scenario approach to optimization which guarantees that each individual oscillator will be well entrained by the resulting stimulus leading to better entrainment at an ensemble level rather than optimizing the waveform for a single representative cell within the population. Allowing for uncertainty in PRCs could be particularly useful in neurons because there is often a great degree of variability in PRCs between samples, as seen in this study as well as in Ota et al. (2011); Wang et al. (2013).

While the numerical methodology developed in this work generates energy-optimal stimuli to achieve entrainment of an ensemble, it is difficult to experimentally prove that a given stimulus is truly optimal. However, the experimental evidence suggests that the resulting stimuli are probably at least close to optimal. Experimental results *in silico* in Section 3 demonstrate that our optimized stimulus resulted in better entrainment in a heterogeneous population of oscillators than other waveforms. We also tested this method *in vitro* using CA1 pyramidal neurons from the hippocampus. In neurons the optimal stimulus performed better than the other suboptimal stimuli with the same power for every cell recorded. This is reassuring because the optimal stimulus is only guaranteed to be optimal for heterogeneity of neurons within an ensemble, but not necessarily for heterogeneity of dynamics within an individual neuron over time. To reconcile the differences between intracellular vs. intercellular heterogeneity, we postulate that the intrinsic properties of the CA1 hippocampal cells, and hence their PRCs, might be slowly changing throughout each trial. Another recent study (Thounaojam et al., 2014) suggested that the natural frequencies of periodically firing neurons can drift over time. A methodology that explicitly accounts for uncertainty in the PRCs across time can help ensure entrainment over the entire duration of the experiment.

In this study, we have performed optimization just for energy minimization but note that strategy could be modified to account for other important constraints by adding terms to the cost function (Equation 22). For example, as we have done in other papers, a cost function could be modified to require charge balanced stimuli (Nabi et al., 2013) or to limit harmful Faradaic reactions (Wilson and Moehlis, 2014b). These and other considerations could be handled on an application specific basis.

A major benefit of this optimization is that it only requires knowledge the phase response properties of an oscillatory system, which can be measured experimentally. The full dynamics of the CA1 hippocampal neuron results from a complex interaction of ionic currents flowing across a cell membrane (Ermentrout and Terman, 2010). While it may one day be possible to estimate the full dynamics of neurons *in vitro* to design an better controller, for example with a Kalman filter (Schiff, 2010; Ullah and Schiff, 2010), phase reduction provides a convenient and experimentally obtainable means of understanding an oscillatory system, even when underlying system dynamics are inaccessible. Furthermore, because the methodology developed in this paper explicitly accounts for heterogeneity in model parameters, different stimuli could be designed accounting for variability across the a small sampling of PRCs.

While this method was tested using electrical stimulation of neurons, the approach could be generalized to many different kinds of stimulation modalities and oscillators. With an optimal stimulation waveform tailored to the dynamics of the system's response to the stimulus, entrainment of the oscillators may be done with greater reliability and less energy than other stimulus waveforms, such as periodic pulsing or sine wave stimulation.

### Conflict of interest statement

The authors declare that the research was conducted in the absence of any commercial or financial relationships that could be construed as a potential conflict of interest.
